# Development and Internal Validation of a Prediction Model for Nasopharyngeal Carcinoma: Using BMI and Inflammatory Response for Deciding Sequence of Chemotherapy

**DOI:** 10.1200/GO.23.00119

**Published:** 2024-02-15

**Authors:** Jiraporn Setakornnukul, Janjira Petsuksiri, Panid Chaysiri, Pongwut Danchaivijitr, Nuttapong Ngamphaiboon, Kullathorn Thephamongkhol

**Affiliations:** ^1^ Division of Radiation Oncology, Department of Radiology, Faculty of Medicine, Siriraj Hospital, Mahidol University, Bangkok, Thailand; ^2^ Division of Medical Oncology, Department of Medicine, Faculty of Medicine, Siriraj Hospital, Mahidol University, Bangkok, Thailand; ^3^ Division of Medical Oncology, Department of Medicine, Faculty of Medicine, Ramathibodi Hospital, Mahidol University, Bangkok, Thailand

## Abstract

**PURPOSE:**

Concurrent chemoradiotherapy followed by adjuvant chemotherapy (CRT-AC) and induction chemotherapy followed by concurrent chemoradiotherapy (IC-CRT) are among the best treatments in nasopharyngeal carcinoma (NPC). This study aimed to develop a model for deciding the sequence of chemotherapy in NPC.

**METHODS:**

Data were separated into two cohorts. The CRT-AC cohort had 295 patients, while the IC-CRT cohort had 112. The predictors were standard factors with BMI and neutrophil-lymphocyte ratio (NLR) to predict overall survival (OS). A flexible parametric survival model was used.

**RESULTS:**

A total of 132 (44.7%) and 72 patients (64.3%) died in the CRT-AC and IC-CRT cohorts, respectively. The predictors in the final models were age, sex, T, N, NLR, and BMI. The models of OS for CRT-AC and IC-CRT had concordance indices of 0.689 and 0.712, respectively, with good calibration curves. When changing the burden of disease along with NLR and BMI, we found that CRT-AC was not significantly different OS from IC-CRT when low NLR (<3) and high burden of disease (T3N3). By contrast, CRT-AC was remarkably more effective when there were high levels of NLR (≥3) and BMI (≥25) with any burden of disease (anyT anyN).

**CONCLUSION:**

With additional BMI and NLR in model, it could be easier to decide between CRT-AC and IC-CRT in countries with limited health care resources.

## Teaser

Consider BMI and NLR with tumor factors in prediction model when choosing treatment sequence for NPC (CRT-AC or IC-CRT).

## INTRODUCTION

About two thirds of new cases of nasopharyngeal carcinoma (NPC) are diagnosed annually in East and Southeast Asia. In Thailand, the age-standardized rate of NPC was 1.7 per 100,000 in 2018 as per the WHO's Global Cancer Observatory.^1^ The evidence indicated that concomitant chemoradiotherapy (CRT) significantly improved overall survival (OS) in locally advanced NPC.^2^

**CONTEXT**

**Key Objective**
The aim of this study was to establish a predictive model aimed at guiding the selection of chemotherapy sequence in individual patients with nasopharyngeal carcinoma (between chemoradiotherapy followed by adjuvant chemotherapy [CRT-AC] and induction chemotherapy followed by concurrent chemoradiotherapy [IC-CRT]).
**Knowledge Generated**
The predictive model is constructed from fundamental prognostic indicators essential for the treatment of nasopharyngeal cancer. These indicators encompass age, sex, T stage, N stage, neutrophil-lymphocyte ratio (NLR), and BMI. Two distinct prediction models, CRT-AC and IC-CRT, were independently formulated. Notably, these models exhibit favorable concordance indices of 0.69 and 0.71, respectively, indicating robust predictive performance. Furthermore, the calibration of the models demonstrates acceptable alignment with observed outcomes.
**Relevance**
In addition to tumor factors, BMI and NLR are pragmatic predictors that help clinicians select the optimal chemotherapy sequence in a broad setting.

Initial studies on adjuvant platinum-based chemotherapy after CRT showed no significant survival benefits.^3-5^ However, recent phase III randomized studies with adjuvant metronomic capecitabine improved survival in patients who completed CRT with or without induction chemotherapy.^6,7^ Therefore, benefit of adjuvant chemotherapy was still controversial. Subsequently, studies on induction chemotherapy followed by concurrent chemoradiotherapy (IC-CRT) consistently showed survival benefits.^8-10^

Until now, there is a lack of direct comparison between concurrent chemoradiotherapy followed by adjuvant chemotherapy (CRT-AC) and IC-CRT.^11^ According to an updated network meta-analysis on NPC, CRT-AC and IC-CRT are among the best treatment options in terms of OS and progression-free survival (PFS), without significant difference between them.^12,13^ Although IC-CRT was the highest-ranking treatment for distant control, it was not included in the three best treatments for locoregional control,^13^ consistent with findings from our retrospective cohort study.^14^ Therefore, the optimal sequences between IC-CRT and CRT-AC remain uncertain for OS and PFS.

In practice, treatment selection relies on tumor stage, determined by clinical trial inclusion criteria. Initial treatment sequences—induction chemotherapy or concurrent chemoradiotherapy—typically have high compliance. However, subsequent treatment poses challenges because of the intense preceding therapy. Randomized control trials generally show average effects. Clinical prediction models aid patient-clinician treatment decisions,^15^ estimating individual probabilities of outcomes such as OS and PFS. NPC prognostic factors include tumor/nodal classification and inflammatory/hematologic factors such as the neutrophil-lymphocyte ratio (NLR) and obesity.^16,17^ Obesity and inflammation strongly associate with cancer mortality.^18^ Despite constant patient and tumor factors, treatment choices can vary on the basis of the clinical prediction model.

This study aimed to create a clinical prediction model that could estimate and compare the OS and PFS achievable with these controversial treatments for locally advanced NPC.

## METHODS

After institutional review board approval, the study population consisted of all consecutive cases of locally advanced NPC (stages II-IVa) patients with histology of WHO types I-III and older than 18 years who were treated with combined intensity-modulated radiotherapy and chemotherapy at Siriraj Hospital, Mahidol University, between January 2007 and December 2014. Patients were excluded if they had other malignancies, had no laboratory data, did not have a curative aim, or were medically unfit for chemotherapy. As there was no routine plasma Epstein-Barr virus (EBV) DNA at our institution, this factor was unavailable. A radiologist restaged all patients on the basis of American Joint Committee on Cancer (AJCC; eighth edition). Given the need to have medically fit patients for chemoradiotherapy, all patients were required to have an Eastern Cooperative Oncology Group performance status of 0 or 1. The protocol for pretreatment evaluation and post-treatment surveillance was added in Appendix 1.

This research is a longitudinal cohort prediction study, which is the natural design of a prognostic prediction study according to the transparent reporting of a multivariable prediction model for individual prognosis or diagnosis (TRIPOD) guideline.^19^ The treatment protocol was added in Appendix 1. The treatment sequence was used to separate the study population into a CRT-AC cohort and an IC-CRT cohort. A prediction model was then developed for each cohort. The two primary clinical outcomes of prediction were standard OS and PFS (Appendix 1). We calculated the follow-up time using the reverse Kaplan-Meier method.^20^

Regarding the prespecified predictor, we classified two aspects. One was patient factors: age at diagnosis, sex, BMI, and NLR. The other was tumor factors: histology, tumor stage, and nodal stage. Age was included in the model as a continuous variable; the categorical variables were BMI with standard classification (underweight/normal/overweight) and NLR with the cutoff of 3.^21^

We used flexible parametric survival models on the development data set to predict OS and PFS by estimating predictor coefficients. The CRT-AC and IC-CRT cohorts were separately analyzed to account for distinct coefficients and treatment interactions.^22,23^ Model performance was assessed individually for discrimination (Harrell's C statistic with 95% CI) and calibration (using PMCALPLOT and STCOXGRP tools with risk groups cut at the 25th and 75th percentiles).^24,25^ Internal validation involved 1,000 bootstrap iterations with the validate function in RMS package^26^ in R software (version 3.9, Vienna, Austria). All model specifications were reported: baseline survival at 5 years, the coefficient of each variable, and the shrinkage factor.

We next simulated the individual prediction demonstrated by the survival curve of nine patients with three different tumor stages (T3N3M0 [high burden of tumor], T3N1M0 [low burden of tumor], and T4N0M0 [high local burden only]). Then, patient predictors, such as NLR and BMI, were varied. The variations were based on (1) low risk of a systemic inflammatory response (SIR; low NLR with normal BMI) and (2) high risk of an SIR (high NLR with overweight, and high NLR with underweight).

### Ethics Approval and Consent to Participate

The study protocol (333/2559 [EC2]) was approved by the Siriraj Institutional Review Board with COA number (Si 502/2016) annually renewed, lastly on September 19, 2023. Written consent requirement was waived due to the study's retrospective nature.

### Consent for Publication

This publication has no identification of any patients.

## RESULTS

**FIG 1:**
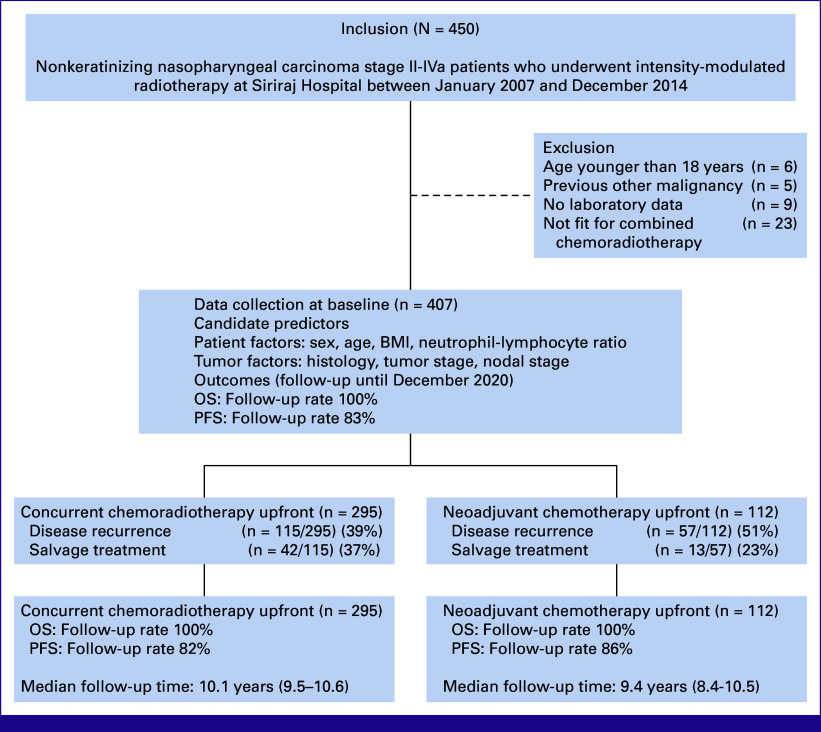
Study flow. OS, overall survival; PFS, progression-free survival.

A total of 450 consecutive patients met the inclusion criteria. Of these, 43 were excluded (Fig 1). The remaining 407 patients were enrolled and separated into two cohorts. One was the CRT-AC cohort (295 of 407 patients [72%]). The second was the IC-CRT cohort (112 of 407 patients [28%]). The extent of weight loss before treatment exceeding 10% was marginal and comparable in both the groups, with 15 of 295 individuals (5.1%) in the CRT-AC cohort and 6 of 112 individuals (5.4%) in the IC-CRT cohort.

**FIG A1:**
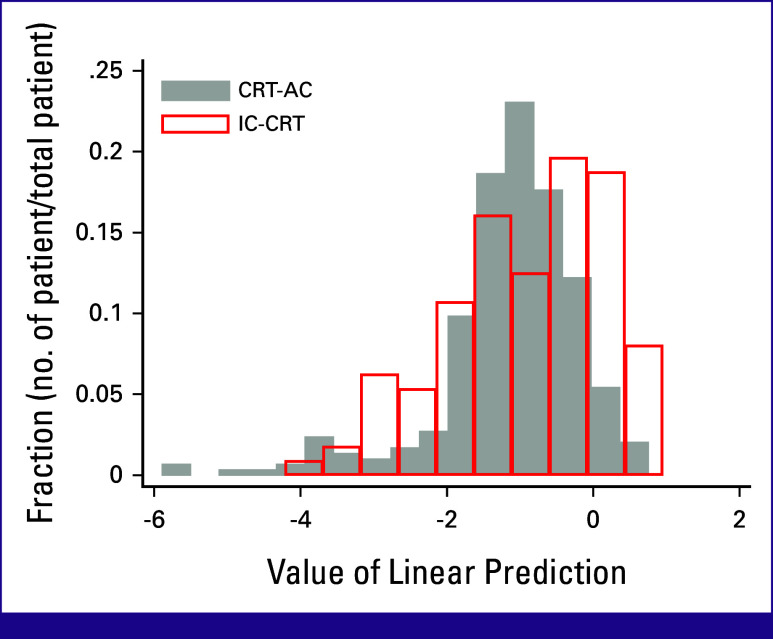
Comparison of the proportion of linear predictor between CRT-AC and IC-CRT cohorts. CRT-AC, chemoradiotherapy followed by adjuvant chemotherapy; IC-CRT, induction chemotherapy followed by concurrent chemoradiotherapy.

A centralized linear predictor distribution showed slightly worse prognosis for the IC-CRT group, especially in higher stage (Table 1, Appendix Fig A1). For this investigation as the compliance with actual practice, 18.6% of patients in the CRT-AC group did not receive adjuvant chemotherapy because of the persistence of severe oral mucositis. Additionally, one patient in the IC-CRT group did not undergo radiotherapy because of experiencing severe fatigue and nausea, leading the patient to decline the treatment. Moreover, 6.3% of patients in the IC-CRT group did not receive concurrent chemotherapy during radiotherapy because of a decline in their performance status and renal function.

**TABLE 1:** Descriptive Patient Characteristics, Treatment, and Outcomes of CRT-AC and IC-CRT, With Univariable Analysis for OS

Factor	Total (N = 407)	CRT-AC (n = 295)	HR (95% CI) of OS	IC-CRT (n = 112)	HR (95% CI) of OS
Sex, No. (%)					
Female	123 (30.2)	95 (32.2)	Ref	28 (25.0)	Ref
Male	284 (69.8)	200 (67.8)	1.15 (0.79 to 1.67)	84 (75.0)	2.33 (1.25 to 4.35)
Age, years					
Median (IQR)	51 (44-58)	52 (44-58)	1.02 (1.01 to 1.04)	49 (41-57)	1.03 (1.01 to 1.05)
Mean (SD)	50.4 (11.8)	51.2 (11.3)		48.3 (12.9)	
BMI, mean (SD)	23.5 (4.1)	23.7 (4.2)	0.98 (0.94 to 1.03)	22.9 (3.6)	1.04 (0.97 to 1.10)
BMI, No. (%)					
<18.5	41 (10.1)	29 (9.8)	Ref	12 (10.7)	Ref
18.5-24.9	234 (57.5)	165 (55.9)	0.66 (0.39 to 1.12)	69 (61.6)	1.18 (0.53 to 2.63)
≥25	132 (32.4)	101 (34.2)	0.61 (0.35 to 1.07)	31 (27.7)	1.83 (0.78 to 4.30)
NLR					
Median (IQR)	2.6 (1.8)	2.5 (1.8-3.6)	1.09 (1.02 to 1.17)	2.7 (1.9-3.8)	1.03 (0.93 to 1.14)
Mean (SD)	3.2 (2.4)	3.2 (2.5)		3.2 (2.0)	
NLR, No. (%)					
<3	261 (64.1)	192 (65.1)	Ref	69 (61.6)	Ref
≥3	146 (35.9)	103 (34.9)	1.24 (0.87 to 1.76)	43 (38.4)	1.82 (1.14 to 2.89)
Histology, No. (%)					
NK-differentiated	144 (35.4)	104 (35.3)	Ref	40 (35.7)	Ref
NK-undifferentiated	263 (64.6)	191 (64.7)	0.66 (0.47 to 0.93)	72 (64.3)	0.75 (0.46 to 1.20)
T stage, No. (%)					
T1	50 (12.3)	41 (13.9)	Ref	9 (8.0)	Ref
T2	135 (33.2)	110 (37.3)	0.92 (0.52 to 1.62)	25 (22.3)	0.71 (0.27 to 1.86)
T3	102 (25.1)	78 (26.4)	1.16 (0.65 to 2.07)	24 (21.4)	1.02 (0.39 to 2.63)
T4	120 (29.5)	66 (22.4)	1.93 (1.09 to 3.41)	54 (48.2)	0.98 (0.41 to 2.31)
N stage, No. (%)					
N0	50 (12.3)	41 (13.9)	Ref	9 (8.0)	Ref
N1	135 (33.2)	110 (37.3)	1.43 (0.56 to 3.64)	25 (22.3)	0.93 (0.38 to 2.31)
N2	102 (25.1)	78 (26.4)	2.14 (0.85 to 5.35)	24 (21.4)	1.08 (0.45 to 2.58)
N3	120 (29.5)	66 (22.4)	3.45 (1.35 to 8.81)	54 (48.2)	2.11 (0.93 to 4.79)
AJCC stage, No. (%)					
II	75 (18.4)	70 (23.7)	Ref	5 (4.5)	Ref
III	126 (31.0)	108 (36.6)	1.47 (0.86 to 2.50)	18 (16.1)	0.85 (0.18 to 4.08)
IVa	206 (50.6)	117 (39.7)	3.01 (1.83 to 4.96)	89 (79.5)	2.40 (0.59 to 9.81)
Treatment, No. (%)					
CRT → AC	239 (58.7)	239 (81.0)	—	0	—
CRT	56 (13.8)	56 (19.0)	—	0	—
IC → CRT	104 (25.6)	0	—	104 (92.9)	—
IC → RT alone	7 (1.7)	0	—	7 (6.3)	—
IC alone	1 (0.2)	0	—	1 (0.9)	—
Outcomes, No. (%)					
Death	204 (50.1)	132 (44.7)	—	72 (64.3)	—
Disease recurrence	172 (42.3)	115 (39.0)	—	57 (50.9)	—
Disease recurrence and/or death	214 (52.6)	140 (47.5)	—	74 (66.1)	—

Abbreviations: CRT-AC, chemoradiotherapy followed by adjuvant chemotherapy; HR, hazard ratio; IC-CRT, induction chemotherapy followed by concurrent chemoradiotherapy; NK, nonkeratinizing cell type; NLR, neutrophil-lymphocyte ratio; OS, overall survival; Ref, reference group; SD, standard deviation.

The median follow-up times (95% CI) were 10.1 (95% CI, 9.5 to 10.5), 10.1 (95% CI, 9.5 to 10.6), and 9.4 (95% CI, 8.4 to 10.5) years for the whole cohort, CRT-AC cohort, and IC-CRT cohort, respectively. Of the 407 enrolled patients, 204 (50.1%) died. More deaths occurred in the IC-CRT cohort (64.3%; 72 of 112 patients) than in the CRT-AC cohort (44.7%; 132 of 295 patients; Table 1).

**FIG A2:**
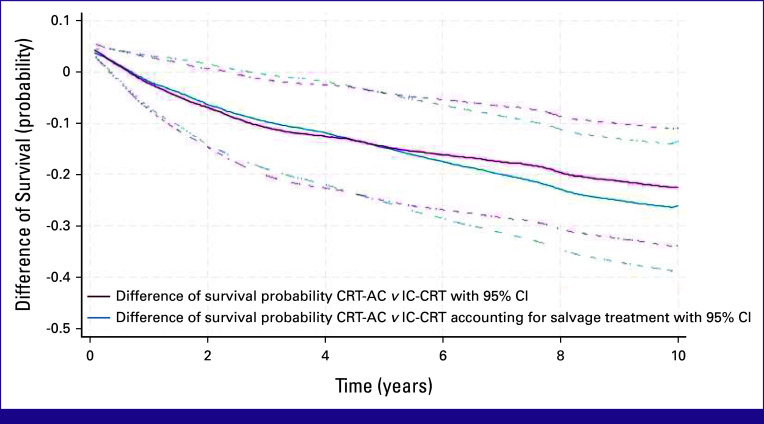
Survival probability difference between CRT-AC and IC-CRT with or without accounting for salvage treatment. CRT-AC, chemoradiotherapy followed by adjuvant chemotherapy; IC-CRT, induction chemotherapy followed by concurrent chemoradiotherapy.

Regarding salvage treatment in our study, the number of patients who underwent salvage treatment did not show a statistically significant difference between the two cohorts (37% in the CRT-AC cohort and 51% in the IC-CRT cohort; *P* = .083; Fig 1). Only one patient in IC-CRT cohort got salvage surgery on his nasopharynx. In the CRT-AC cohort, 19 of 115 patients (17%) received reirradiation for locoregional recurrence, while 20% received systemic chemotherapy. Conversely, within the IC-CRT cohort, 3 of 57 patients (5%) underwent local surgery and/or reirradiation for locoregional recurrence, with 18% choosing systemic treatment. No patients received salvage immunotherapy or targeted therapy. When the survival analysis was performed by censoring patients who had salvage treatment, the Kaplan-Meier survival curve did not differ significantly from the survival curve that did not include censoring because of salvage treatment (Appendix Fig A2).

There was no significant difference in the proportions of patients in the CRT-AC and IC-CRT groups who completed the course of radiotherapy and had a median radiotherapy dose of 6,996 cGy (96.6% and 93.7%, respectively; Appendix Table A1). Nonetheless, there was a statistically significant difference related to the chemotherapy of the cohorts (Appendix Table A1).

The 5-, 8-, and 10-year OS and PFS rates of the IC-CRT cohort were lower than those of the CRT-AC cohort (Appendix Table A2). A univariable analysis found that age, tumor stage, and nodal stage were statistically significant for OS in both cohorts. However, histology was significant only in the CRT-AC cohort, while sex and NLR were significant only in the IC-CRT cohort (Table 1). We decided to enter all these factors to build the final model.

The predictors of the final prediction model for OS and PFS were composed of patient and tumor factors. The patient factors were age at diagnosis of NPC, sex, BMI, and NLR before treatment. The tumor factors were histology, tumor stage, and nodal stage. Notably, the adjusted analysis of patient factors differed for both the OS and PFS of the CRT-AC and IC-CRT groups, particularly for BMI and NLR. The analyses were reported as hazard ratios (HRs; Table 2) and coefficients in the model specification (Table 3). The HR for the CRT-AC group was nearly similar to that of the whole cohort (Appendix Table A3). In the case of tumor factors, their effect sizes for the CRT-AC and IC-CRT groups were nearly similar. However, the tumor factors were slightly more significant in the CRT-AC group than in the IC-CRT group because of the former's higher number of patients.

**TABLE 2:** Separate Prediction Model of CRT-AC and IC-CRT With Discrimination, Internal Validation, and Shrinkage Factor for OS and PFS

Factor	OS				PFS			
	CRT-AC (n = 295)		IC-CRT (n = 112)		CRT-AC (n = 295)		IC-CRT (n = 112)	
	HR (95% CI)	*P*	HR (95% CI)	*P*	HR (95% CI)	*P*	HR (95% CI)	*P*
Sex								
Female	Ref		Ref		Ref		Ref	
Male	1.01 (0.68 to 1.49)	.958	2.34 (1.22 to 4.51)	0.011	0.96 (0.66 to 1.38)	.812	2.30 (1.23 to 4.30)	.009
Age, years	1.03 (1.02 to 1.05)	<.001	1.03 (1.01 to 1.05)	0.013	1.02 (1.01 to 1.04)	.002	1.02 (1.00 to 1.05)	.035
BMI								
<18.5	Ref		Ref		Ref		Ref	
18.5-24.9	0.86 (0.49 to 1.52)	.608	1.07 (0.45 to 2.51)	.883	1.03 (0.58 to 1.81)	.922	1.18 (0.50 to 2.81)	.702
≥25	0.79 (0.43 to 1.45)	.446	2.12 (0.83 to 5.41)	.118	0.91 (0.49 to 1.66)	.752	2.05 (0.76 to 5.49)	.155
NLR								
<3	Ref		Ref		Ref		Ref	
≥3	1.15 (0.79 to 1.68)	.455	2.22 (1.28 to 3.85)	.004	1.26 (0.88 to 1.81)	.214	2.14 (1.25 to 3.65)	.006
Histology								
NK-diff	Ref		Ref		Ref		Ref	
NK-undiff	0.61 (0.42 to 0.87)	.007	0.81 (0.49 to 1.32)	.395	0.59 (0.42 to 0.84)	.004	0.90 (0.55 to 1.47)	.673
T stage								
T1	Ref		Ref		Ref		Ref	
T2	0.83 (0.47 to 1.48)	.524	0.99 (0.34 to 2.94)	.990	0.80 (0.46 to 1.38)	.417	1.15 (0.40 to 3.28)	.790
T3	1.08 (0.60 to 1.96)	.796	1.48 (0.51 to 4.33)	.472	0.98 (0.56 to 1.74)	.956	1.56 (0.54 to 4.46)	.410
T4	2.08 (1.16 to 3.74)	.014	1.68 (0.61 to 4.65)	.319	1.96 (1.12 to 3.44)	.019	1.64 (0.61 to 4.45)	.328
N stage								
N0	Ref		Ref		Ref		Ref	
N1	1.50 (0.58 to 3.92)	.403	1.00 (0.38 to 2.63)	.992	1.34 (0.52 to 3.48)	.547	1.01 (0.38 to 2.67)	.995
N2	2.46 (0.96 to 6.28)	.060	1.25 (0.50 to 3.01)	.636	2.59 (1.02 to 6.58)	.045	1.10 (0.45 to 2.74)	.830
N3	4.58 (1.76 to 11.94)	.002	3.58 (1.42 to 9.02)	.007	4.40 (1.69 to 11.43)	.002	2.84 (1.14 to 7.03)	.024
Original C-index (95% CI)	68.91 (64.40 to 73.43)		71.24 (65.15 to 77.34)		68.02 (63.69 to 72.37)		69.19 (62.94 to 75.42)	
After internal validation	65.62 (61.14 to 70.09)		66.52 (60.50 to 72.53)		64.97 (60.75 to 69.19)		63.94 (57.85 to 70.03)	
Shrinkage factor (SD)	0.80 (0.12)		0.69 (0.14)		0.80 (0.12)		0.68 (0.14)	

Abbreviations: C-index, concordance index; CRT-AC, chemoradiotherapy followed by adjuvant chemotherapy; HR, hazard ratio; IC-CRT, induction chemotherapy followed by concurrent chemoradiotherapy; N, node; NLR, neutrophil-lymphocyte ratio; OS, overall survival; PFS, progression-free survival; Ref, reference group; SD, standard deviation; T, tumor.

**TABLE 3:** Model Specification of Concurrent CRT-AC and IC-CRT

Model	Calculation Formula
Concurrent chemoradiotherapy followed by adjuvant chemotherapy (CRT-AC)	
OS	Baseline OS at fifth year (S0_5yr) = 0.959645 Shrinkage factor = 0.7982293 LP = 0.0314288 × Age + (–0.1873802) × T2 + 0.0784666 × T3 + 0.7345331 × T4 + 0.4084085 × N1 + 0.8997947 × N2 + 1.522467 × N3 + 0.1436826 × (NLR ≥3) + 0.0103304 × Male + (–0.4973013) × Undiff histology + (–0.1486681) × (BMI 18.5-24.9) + (–0.2378402) × (BMI ≥25) 5-year OS probability = (S0_5yr^ exp [LP × Shrinkage factor])
PFS	Baseline PFS at fifth year (S0_5yr) = 0.92757586 Shrinkage factor = 0.796134 LP = 0.0244771 × Age + (–0.2268772) × T2 + (–0.0159433) × T3 + 0.6729845 × T4 + 0.2930496 × N1 + 0.9529436 × N2 + 1.481684 × N3 + 0.2297136 × (NLR ≥3) + (–0.0448531) × Male + (–0.52055) × Undiff histology + (0.0281928) × (BMI 18.5-24.9) + (–0.0978997) × (BMI ≥25) 5-year PFS probability = (S0_5yr^ exp [LP × Shrinkage factor])
Induction chemotherapy followed by concurrent chemoradiotherapy (IC-CRT)	
OS	Baseline overall survival at fifth year (S0_5yr) = 0.98155319 Shrinkage factor = 0.6852513 LP = 0.0297141 × Age + (–0.0067188) × T2 + 0.3935126 × T3 + 0.5177233 × T4 + (–0.0049467) × N1 + 0.2194883 × N2 + 1.275791 × N3 + 0.7973309 × (NLR ≥3) + 0.8520867 × Male + (–0.2141256) × Undiff histology + 0.064314 × (BMI 18.5-24.9) + 0.749097 × (BMI ≥25) 5-year OS probability = (S0_5yr^ exp [LP × Shrinkage factor])
PFS	Baseline PFS at fifth year (S0_5yr) = 0.96319809 Shrinkage factor = 0.6831365 LP = 0.0230992 × Age + (0.1422841) × T2 + (0.4427307) × T3 + 0.4966272 × T4 + 0.0030721 × N1 + 0.0996469 × N2 + 1.042845 × N3 + 0.7593933 × (NLR ≥3) + (0.8327601) × Male + (–0.1053996) × Undiff histology + (0.1690173) × (BMI 18.5-24.9) + (0.7154723) ×(BMI ≥25) 5-year PFS probability = (S0_5yr^ exp [LP × Shrinkage factor])

Abbreviations: CRT-AC, chemoradiotherapy followed by adjuvant chemotherapy; IC-CRT, induction chemotherapy followed by concurrent chemoradiotherapy; LP, linear predictor; NLR, neutrophil-lymphocyte ratio; OS, overall survival; PFS, progression-free survival; Undiff, undifferentiated.

**FIG 2:**
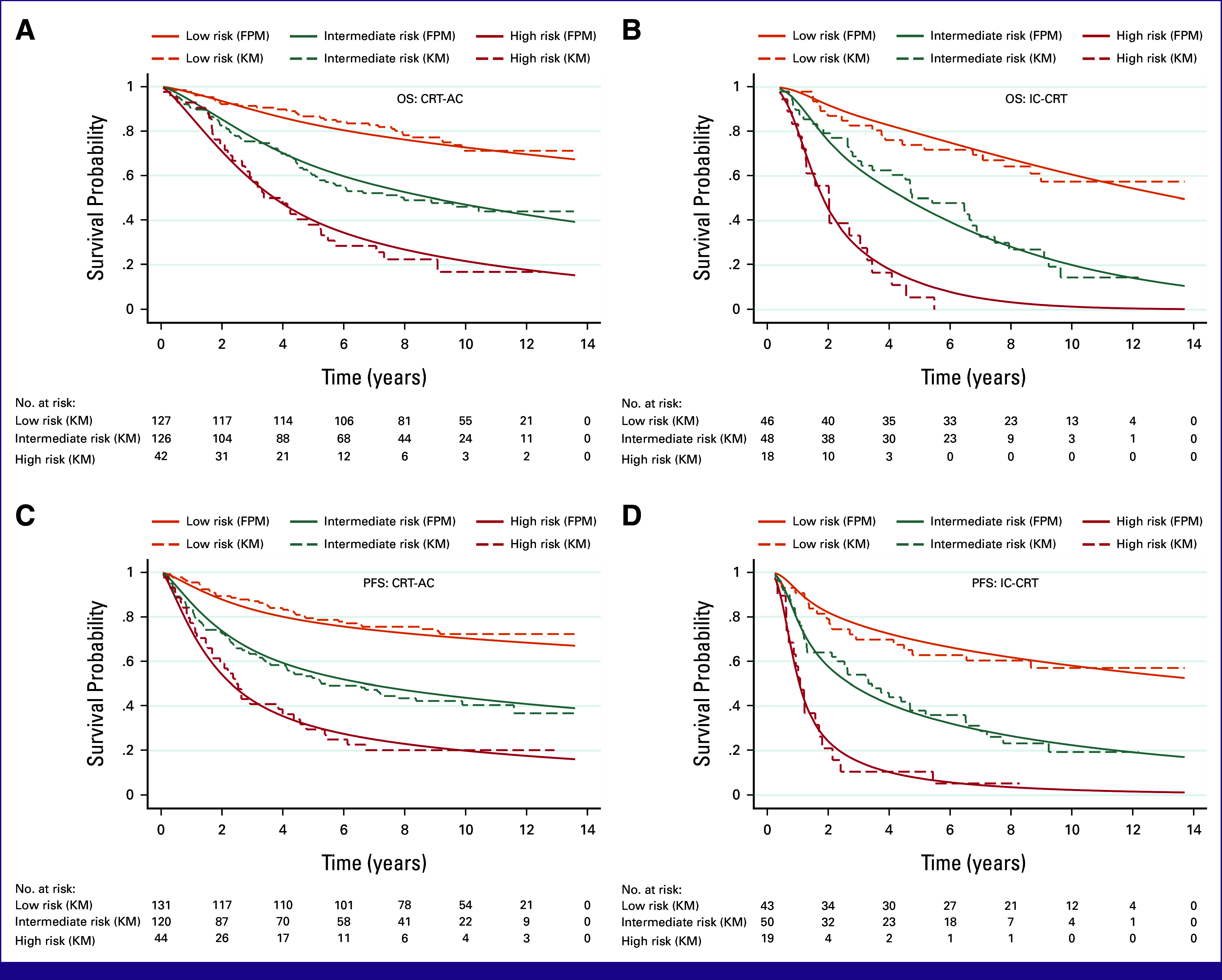
Calibration plot of FPM. Solid lines are survival probability from FPM and dotted lines are survival probability from Kaplan-Meier curve. (A) OS of CRT-AC cohort. (B) OS of IC-CRT cohort. (C) PFS of CRT-AC cohort. (D) PFS of IC-CRT cohort. CRT-AC, chemoradiotherapy followed by adjuvant chemotherapy; FPM, flexible parametric survival model; IC-CRT, induction chemotherapy followed by concurrent chemoradiotherapy; KM, Kaplan-Meier curve; OS, overall survival; PFS, progression-free survival.

**FIG A3:**
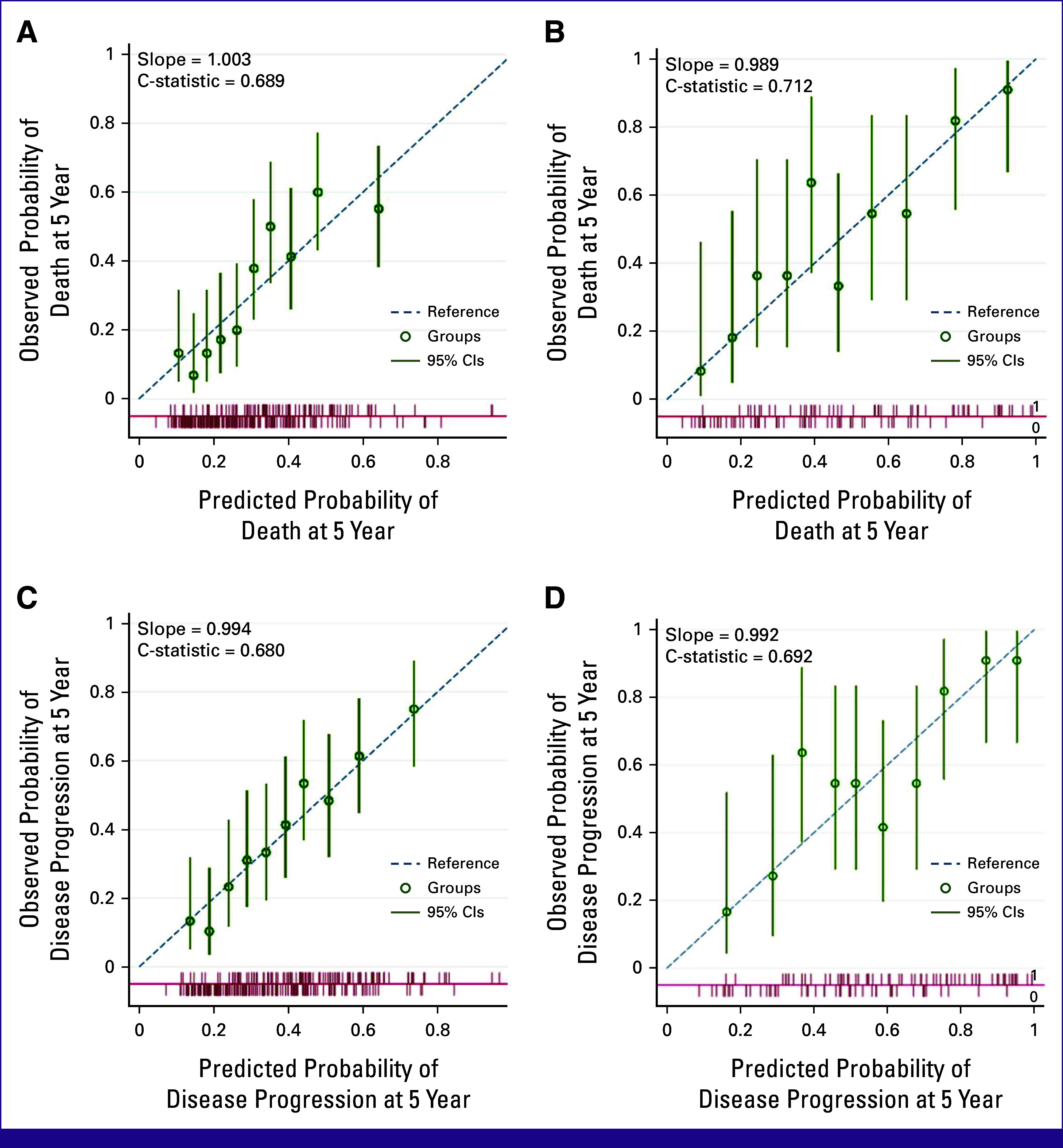
Calibration plot at 5-year outcome. (A) OS of CRT-AC cohort. (B) OS of IC-CRT cohort. (C) PFS of CRT-AC cohort. (D) PFS of IC-CRT cohort. CRT-AC, chemoradiotherapy followed by adjuvant chemotherapy; IC-CRT, induction chemotherapy followed by concurrent chemoradiotherapy; OS, overall survival; PFS, progression-free survival.

The prediction model's performance was reported as discrimination and calibration. First, the discrimination of the prediction model of the CRT-AC cohort was shown as the concordance index (C-index). The values for OS and PFS were 68.91 (95% CI, 64.40 to 73.43) and 68.02 (95% CI, 63.69 to 72.37), respectively (Table 2). The C-index of the IC-CRT cohort was slightly higher: OS, 71.24 (95% CI, 65.15 to 77.34) and PFS, 69.19 (95% CI, 62.94 to 75.42). The C-index of the whole cohort is shown in Appendix Table A3. Second, the calibration plot of the 5-year OS and PFS of the CRT-AC and IC-CRT cohorts showed generally good calibration (Appendix Fig A3). In the same pattern, the whole-curve calibration plot shows minimal underestimation and overestimation for both CRT-AC and IC-CRT (Fig 2).

After bootstrapping for internal validation of OS and PFS, the C-index changed slightly, falling by approximately 3% for the CRT-AC cohort and 5% for the IC-CRT cohort (Table 2). The model specification is presented in Table 3. The mean and proportion of the linear predictors from the prediction model of the IC-ACR cohort were higher than those of the CRT-AC cohort (–0.92 *v* –1.15, respectively; Appendix Table A1). For the CRT-AC cohort, the shrinkage factor was 0.8 each for OS and PFS. Regarding the IC-CRT cohort, the shrinkage factor was 0.69 and 0.68 for OS and PFS, respectively (Table 3).

**FIG A4:**
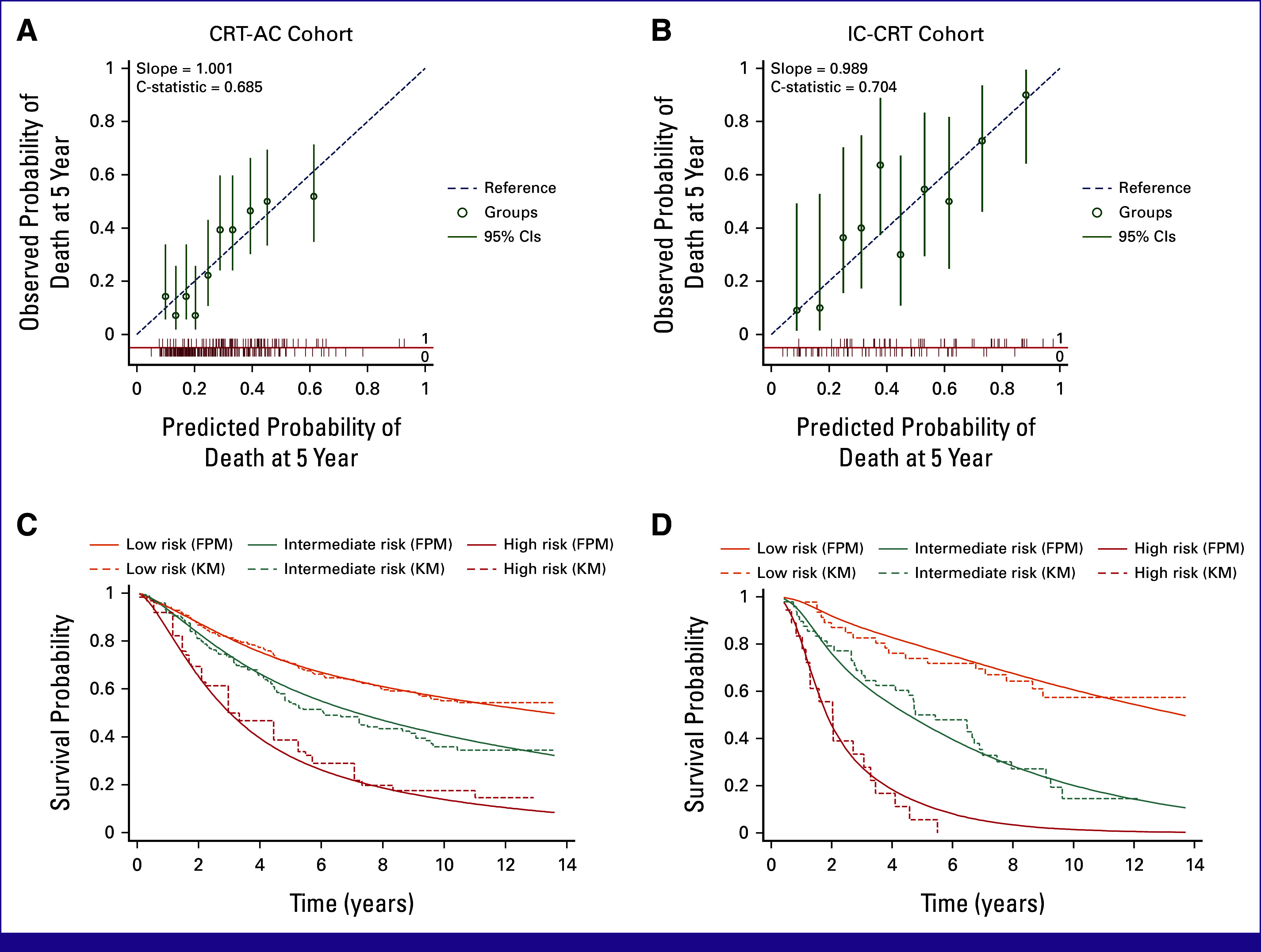
Calibration for sensitivity analysis. (A) Calibration plot at 5-year OS for CRT-AC. (B) Calibration plot at 5-year OS for IC-CRT. (C) Calibration plot of OS curve for CRT-AC. (D) Calibration plot of OS curve for IC-CRT. CRT-AC, chemoradiotherapy followed by adjuvant chemotherapy; FPM, flexible parametric survival model; IC-CRT, induction chemotherapy followed by concurrent chemoradiotherapy; KM, Kaplan-Meier curve; OS, overall survival.

A sensitivity analysis was conducted on patients eligible for cisplatin chemotherapy. Among these, 278 patients received cisplatin in the CRT-AC cohort, whereas 105 patients were administered cisplatin either induction or concurrent chemotherapy in the IC-CRT cohort. The C-index of the original model and the model after internal validation exhibited similarity to those of the entire population, which encompassed both cisplatin and carboplatin chemotherapy patients, in both cohorts (Appendix Table A4). Likewise, the calibration was favorable for both at the 5-year calibration plot and the whole-curve calibration plot (Appendix Fig A4).

**FIG 3:**
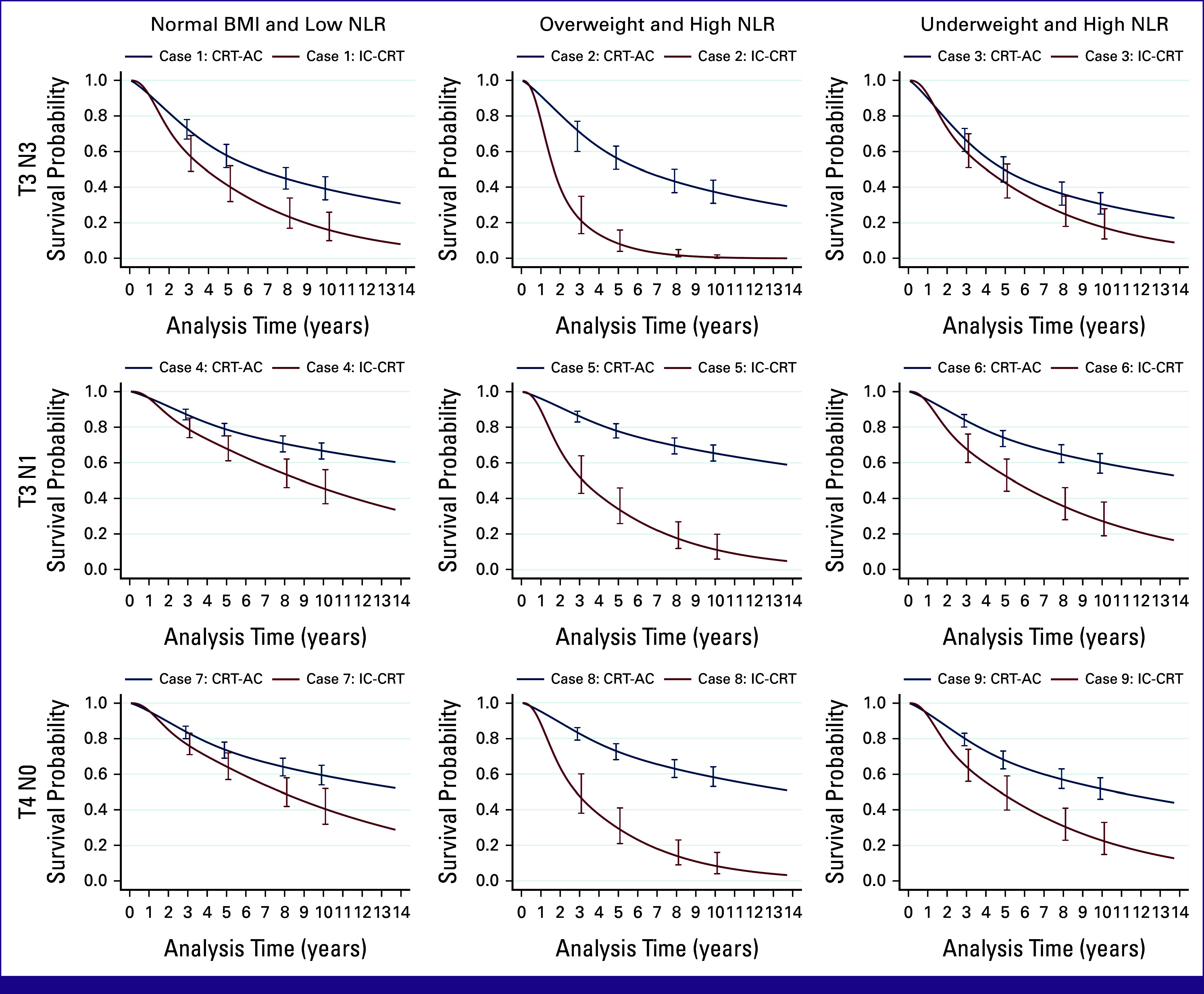
Example cases of individual prediction comparing between CRT-AC (navy blue) and IC-CRT (maroon). CRT-AC, chemoradiotherapy followed by adjuvant chemotherapy; IC-CRT, induction chemotherapy followed by concurrent chemoradiotherapy; N, node; NLR, neutrophil-lymphocyte ratio; T, tumor.

Turning to the case scenarios, scenarios 1-3 had identical tumor factors (high T stage and N stage [T3N3M0]). However, these case scenarios had different SIRs: scenario 1, low SIR; scenario 2, high SIR with overweight; and scenario 3, high SIR with underweight (Fig 3, Appendix Table A5). In scenarios 1 (low SIR) and 3 (high SIR with underweight), IC-CRT did not have significant difference of 5-year OS when compared with CRT-AC. However, the 5-year OS for IC-CRT was significantly decreased in case 2 (high SIR with overweight; Fig 3 and Appendix Table A5).

Case scenarios 4-6 had a low disease burden (T3N1M0). In the same way, the 5-year OS for IC-CRT was significantly decreased, especially in case scenario 5 (high SIR with overweight). Likewise, IC-CRT had a slightly lower 5-year OS than CRT-AC when there was a low SIR (scenario 4). This poor 5-year OS was also clearly demonstrated in T4N0 with high SIR with overweight (scenario 8). In summary, the 5-year OS for IC-CRT was remarkably decreased in all scenarios of high SIR with overweight (Fig 3 and Appendix Table A5).

## DISCUSSION

To assess individual survival of locally progressed NPC patients, two clinical prediction models were developed: CRT-AC and IC-CRT. Both versions performed well in discriminating and calibration tests. OS C-statistics were 0.689 in the CRT-AC model and 0.712 in the IC-CRT model; PFS was 0.680 and 0.692, respectively. The whole-curve calibration matched the Kaplan-Meier estimate well. The models were bootstrapped for internal validation. We used the shrinkage factor to quantify overfitting and correct the models. Furthermore, most patients in the IC-CRT and CRT-AD groups received platinum-fluorouracil (FU), with carboplatin-treated individuals excluded for sensitivity analysis. This ensured that diverse regimens had no impact on induction and adjuvant chemotherapy within our study. Regarding our study population, we simulated the use of these prediction models before treatment initiation in separate cohorts. This aligns with the intention-to-treat principle, as patient adherence to treatment remains uncertain at this stage. Our prediction model remains valid during the prediction time frame, regardless of eventual treatment adherence. In terms of patient compliance, 19% of CRT-AC cohort patients did not receive adjuvant chemotherapy, and 6% of IC-CRT patients did not undergo concurrent treatment because of treatment-related toxicity. These rates mirror real-world compliance patterns. Similarly, the Forum for Nuclear Cooperation in Asia (FNCA) study showed that 15% of CRT-AC patients could not undergo adjuvant chemotherapy, while ongoing FNCA research on induction chemotherapy followed by CRT indicated a 4% inability to proceed with concurrent treatment, aligning with our findings.^27^

The two models clearly showed different effects of some variables. For example, NLR and BMI were more highly associated with death in the IC-CRT model than in the CRT-AC model (Table 2). However, the T stage and N stage effects were less associated with death in the IC-CRT model than in the CRT-AC model. Such differences generated different individual predictions of outcomes from treatment effects (Fig 3). This treatment effect is a challenging issue to address in clinical prediction models, and it is similar to the problems encountered in studies of causal inference. If we put the treatment option into the model, it would be under the assumption that all variables would have the same effect between treatments.

Consequently, our study was performed with two prediction models.^22,23^ One was for CRT-AC, and the other was for IC-CRT. We aimed to allow the independent coefficients of the variables and support the interaction effects among the treatments. Before testing the model performance, we checked the overlap of the linear predictors of the two cohorts (CRT-AC and IC-CRT). IC-CRT's linear predictor had a higher proportion of poor prognoses than CRT-AC's predictor, and most of the prognoses overlapped (Appendix Fig A1).

In our platinum-based chemotherapy study, 6% of patients in both the CRT-AC and IC-CRT cohorts received carboplatin. Although carboplatin is typically less potent as a radiosensitizer than cisplatin in ovarian cancer, a systematic review in nasopharyngeal cancer demonstrated comparable survival rates for both.^28^ Furthermore, this was supported by the noninferiority trial by Chitapanarux.^29^ Notably, the sensitivity analysis on cisplatin-treated patients in our study, the two critical measurements of model performance indicated very similar model performance to the whole population in both cohorts. The first critical measurement was the C-index for CRT-CA (68.48 *v* 68.91), and for IC-CRT C-index (70.43 *v* 71.24). The second one was the calibration curves, which aligned closely with the original population (Appendix Table A4 and Fig A4). For these reasons, our prediction models are applicable to patients receiving either cisplatin or carboplatin treatments.

The patients with NPC who were obese had significantly poorer survival outcomes in the IC-CRT model, when compared with the CCRT-AC model. There are several reasons for this finding. First, the ASCO guidelines recommend full weight-based chemotherapy dosing for curative aims regardless of obesity. Nevertheless, few patients with obesity receive chemotherapy dosing calculated using their actual body weight in practice.^30^ Insufficient chemotherapy dosing may cause inadequate tumoricidal effects and delay cancer treatment.^31^ Second, patients who are obese have a high proportion of fat. The fat modulates renal and liver functions related to drug metabolism and drug excretion and is also associated with chronic inflammation. The high proportion of fat might lead to altered pharmacokinetics and impaired drug delivery. These adverse changes result in obesity-associated chemoresistance.^32^ Therefore, patients with NPC who are obese should start treatment with radiotherapy component to avoid local treatment delay and to avoid obesity-associated chemoresistance.

Our patient population for the prediction model included stage II NPC. With the eighth edition of the AJCC Staging System, tumors involving the medial/lateral pterygoid muscle or prevertebral muscle were moved from T4 disease to T2 disease.^33^ Thus, in patients with a low nodal disease burden, such as N0-1, the cancer stage was downstaged from stages III-IVa to stage II NPC. However, this kind of patient was included in a landmark phase III randomization trial.^34^ In the current investigation, stage II patients who had bulky tumor disease were treated with concurrent chemoradiotherapy with adjuvant chemotherapy or neoadjuvant chemotherapy. Our prediction model can be used for patients with stage II NPC who have bulky primary tumors or bulky lymph nodes planned to be treated with adjuvant chemotherapy or neoadjuvant chemotherapy.

The predictors in our model were tumor factors (tumor stage, nodal stage, and histology) and patient factors (sex, age, BMI, and NLR). Our prediction model did not include plasma EBV DNA because we did not have this information in the retrospective data. A previous publication of a prediction model for NPC in which plasma EBV DNA was one of the predictors showed a higher C-index (range, 0.72-0.80) than for our prediction model.^35,36^ There are some limitations of plasma EBV DNA in terms of prognostication; for example, one quarter of endemic nasopharyngeal cancer was undetectable plasma EBV DNA.^37^ In addition, there are many heterogeneities in each process of plasma EBV DNA testing and variations in its cutoffs, which range from 1,500 to 25,000 copies/mL.^38^ If plasma EBV DNA is one of the predictors, standardization and external validation in different laboratories are needed.^39^

For clinical applications of these prediction models, we will simulate patients with varying tumor stages, NLR values, and BMI statuses, as detailed in Appendix Table A5. Individual survival curves generated by the prediction models are presented for each patient in Figure 3. This information is invaluable for informed decision making. For instance, in case scenarios 2, 5, and 8, where patients were overweight with high NLR levels, CRT-AC demonstrated a significant superiority over IC-CRT, making CRT-AC the preferred treatment option. However, in situations where the survival outcomes did not exhibit significant differences because of overlapping 95% CIs, both IC-CRT and CRT-AC should be considered as treatment options.

This study has a few limitations. First, there were missing data, consistent with the nature of retrospective cohort studies. The absence of data may cause overestimation or underestimation by the prediction model. Second, we need to validate these results using another data set before the model is put to clinical use. Third, the applicability of our findings might be limited by the utilization of cisplatin/carboplatin-FU in the IC-CRT cohort. However, a strength of the study is its adequate long-term follow-up period. We also performed a flexible parametric model, which was very adaptive for calibration. As a result, our prediction is accurate according to the whole-curve calibration. These new simplified prediction models would prove immensely valuable and pragmatic for applications in settings with limited resources, where there is no requirement for plasma EBV DNA laboratory testing.

In conclusion, our results showed the different effects of BMI and NLR on the timing of chemotherapy. To our knowledge, this is the first study to report this finding. Drawing upon these differences, we have developed a clinical prediction model for each treatment approach (IC-CRT or CRT-AC). These prediction models can help doctors and patients with their decision making. External validation is needed to confirm our prediction models.

## SUPPORT

Supported by the Thailand Grand Challenge Program for Research University Network (RUN) under the Precision Medicine for Cancer project by the National Research Council of Thailand (NRCT) and was also funded by Siriraj Research Development Fund (managed by Routine to Research: R2R).

## DATA SHARING STATEMENT

The data, Stata, and R code that support the findings of this study are available from the corresponding author upon reasonable request.

## APPENDIX 1. SUPPLEMENT MATERIALS AND METHODS

### Pretreatment Evaluation and Posttreatment Surveillance

For pretreatment evaluation, all enrolled patients underwent an ENT physical examination, transnasal endoscopy, and either computed tomography (CT) of the head and neck or magnetic resonance imaging of the nasopharynx and neck for primary tumor and nodal staging. As part of the systemic workup, a chest x-ray or CT of the chest was performed to evaluate lung metastasis, while ultrasonography or CT of the upper abdomen was conducted to assess liver metastasis. Additionally, a bone scan was done to evaluate bone metastasis.

After completing the full course of treatment, all patients underwent treatment response evaluation at 12-20 weeks, which involved transnasal endoscopy and nasopharyngeal biopsy conducted by an otolaryngologist, along with imaging of the nasopharynx and neck using CT scan or magnetic resonance imaging. Routine follow-up visits consisted of ENT examinations every 2-4 months during the first 2 years, and later, every 4-6 months. As for imaging surveillance, periodic CT scans or magnetic resonance imaging was conducted occasionally.

### Treatment Protocol

During the study period, concurrent chemoradiotherapy followed by adjuvant chemotherapy (CRT-AC) was the standard of care at our institute for locally advanced nasopharyngeal carcinoma (NPC). This regimen was provided as a matter of routine management, and it was in line with the recommendations of Intergroup Study 0099 (20). Occasionally, some patients with bulky cervical lymph nodes (N3 disease) or intracranial extension of the nasopharyngeal mass (T4 disease) were selected for treatment with induction chemotherapy followed by concurrent chemotherapy (IC-CRT). For stage II NPC, some patients with bulky nodal disease (particularly lymph node sizes exceeding 3 cm) were treated with concurrent chemoradiotherapy with adjuvant or neoadjuvant chemotherapy. Intensity-modulated radiotherapy with a simultaneous integrated boost with 6,996 cGy in 33 fractions to a high-risk target volume was the standard radiotherapy treatment. Patients received concurrent chemotherapy with cisplatin (100 mg/m^2^ once every 3 weeks). Cisplatin plus fluorouracil for adjuvant chemotherapy or induction chemotherapy was the routine regimen. However, carboplatin was given instead of cisplatin if a patient had renal insufficiency.

### Sample Size Estimation

The estimation of the sample size was based on at least 10 events per variable. As we had seven potentially selected variables, at least 70 events were needed for each model to achieve enough statistical power recommended by transparent reporting of a multivariable prediction model for individual prognosis or diagnosis (TRIPOD) statement.

### Outcome Definition

The two primary clinical outcomes of prediction were standard OS and progression-free survival (PFS). These were defined as the time from the first treatment date until the date of an event. The first treatment date could be either day 1 of the radiotherapy course or the date of the first cycle of induction chemotherapy. For OS, the event was death from any cause. We calculated the follow-up time using the reverse Kaplan-Meier method. For PFS, the event was disease recurrence or death from any cause. Disease recurrence refers to tumor progression after treatment, encompassing both locoregional disease and distant metastases. Instances where the tumor remained stable or or had a partial response after treatment were not classified as disease recurrence.

**TABLE A1:** Treatment Compliance

Factor	CRT-AC (n = 295)	IC-CRT (n = 112)	*P*
CMT regimen during CCRT			
Regimens, No. (%)			
Carboplatin once a week	17 (5.8)	22 (21.2)	.009
Cisplatin once every 3 weeks	278 (94.2)	82 (78.8)	
No. of cycles of carboplatin, median (IQR)	5 (5-6)	4 (3-5)	.0183
No. of cycles of cisplatin, (%)			
No CCRT	—	8 (8.9)	<.001
1 cycle	63 (22.7)	15 (16.7)	
2 cycles	142 (51.1)	52 (57.8)	
3 cycles	73 (26.3)	15 (16.7)	
Induction/adjuvant chemotherapy, No. (%)			
Regimens			
Cisplatin/carboplatin-FU	240 (100)	109 (97.3)	.032
Others	—	3 (2.7)	
No. of chemotherapy cycles			
No induction/adjuvant chemotherapy	55 (18.7)	—	<.001
1 cycle	29 (9.8)	4 (3.6)	
2 cycles	31 (10.5)	15 (13.4)	
3 cycles	180 (61.0)	93 (83.0)	
Radiotherapy			
Completion of radiotherapy, No. (%)			
Yes	285 (96.6)	105 (93.7)	.264
No	10 (3.4)	7 (6.3)	
Radiation dose, cGy, median (IQR)			
Completion of radiotherapy	6,996 (6,996-6,996)	6,996 (6,996-6,996)	.526
Incompletion of radiotherapy	4,346 (3,392-5,088)	4,876 (3,900-6,360)	.639

Abbreviations: CCRT, concurrent chemoradiotherapy; CMT, chemotherapy; CRT-AC, chemoradiotherapy followed by adjuvant chemotherapy; IC-CRT, induction chemotherapy followed by concurrent chemoradiotherapy; FU, fluorouracil; NK, nonkeratinizing cell type.

**TABLE A2:** A 3-Year, 5-Year, 8-Year, and 10-Year Overall Survival and PFS of CRT-AC and IC-CRT

Year of Outcome	Survival Probability (95% CI)	
	CRT-AC	IC-CRT
OS, years		
3	80.0 (74.6 to 83.8)	67.9 (58.4 to 75.6)
5	68.5 (62.8 to 73.4)	52.7 (43.1 to 61.4)
8	57.9 (52.0 to 63.4)	38.4 (29.1 to 47.6)
10	52.9 (46.6 to 58.8)	30.6 (21.3 to 40.4)
PFS, years		
3	70.5 (65.0 to 75.4)	52.7 (43.1 to 61.4)
5	61.0 (55.2 to 66.3)	42.9 (33.6 to 51.8)
8	54.3 (48.4 to 59.8)	34.9 (26.1 to 43.9)
10	51.7 (45.6 to 57.4)	31.9 (23.0 to 41.1)

Abbreviations: CRT-AC, chemoradiotherapy followed by adjuvant chemotherapy; IC-CRT, induction chemotherapy followed by concurrent chemoradiotherapy; OS, overall survival; PFS, progression-free survival.

**TABLE A3:** Prediction Model of Whole Cohort (n = 407) With Discrimination, Internal Validation, and Shrinkage Factor for OS and PFS

Factor	OS		PFS	
	HR (95% CI)	*P*	HR (95% CI)	*P*
Sex				
Female	Ref		Ref	
Male	1.27 (0.91 to 1.75)	.155	1.23 (0.90 to 1.68)	.192
Age, years	1.03 (1.02 to 1.04)	<.001	1.02 (1.01 to 1.03)	<.001
BMI				
<18.5	Ref		Ref	
18.5-24.9	0.89 (0.56 to 1.40)	.612	1.02 (0.65 to 1.61)	.917
≥25	0.97 (0.59 to 1.59)	.897	1.06 (0.65 to 1.74)	.816
NLR				
<3	Ref		Ref	
≥3	1.34 (1.00 to 1.80)	.053	1.42 (1.07 to 1.89)	.017
Histology				
NK-diffentiated	Ref		Ref	
NK-undifferentiated	0.69 (0.52 to 0.92)	.011	0.70 (0.53 to 0.93)	.014
T stage				
T1	Ref		Ref	
T2	0.88 (0.53 to 1.44)	.604	0.89 (0.55 to 1.43)	.632
T3	1.23 (0.74 to 2.04)	.424	1.16 (0.71 to 1.90)	.548
T4	2.21 (1.36 to 3.59)	.001	2.07 (1.30 to 3.31)	.002
N stage				
N0	Ref		Ref	
N1	1.26 (0.67 to 2.38)	.479	1.13 (0.60 to 2.13)	.707
N2	1.90 (1.02 to 3.55)	.044	1.89 (1.01 to 3.53)	.044
N3	4.26 (2.25 to 8.05)	<.001	3.87 (2.05 to 7.30)	<.001
C-index (95% CI)	69.21 (65.58 to 72.85)		67.97 (64.49 to 71.44)	
Internal validation (95% CI)	67.14 (63.75 to 70.53)		65.84 (62.60 to 69.07)	
Shrinkage factor (SD)	0.87 (0.09)		0.86 (0.09)	

Abbreviations: C-index; concordance index; HR; hazard ratio; N, node; NK, nonkeratinizing cell type; NLR, neutrophil-lymphocyte ratio; OS, overall survival; PFS, progression-free survival; Ref, reference group; SD, standard deviation; T, tumor.

**TABLE A4:** Sensitivity Analysis of Model Performance

Sensitivity Analysis (OS)	CRT-AC (only cisplatin) N = 278	IC-CRT (only cisplatin) N = 105
Original C-index (95% CI)	68.48 (63.60 to 73.37)	70.43 (63.99 to 76.86)
After internal validation (95% CI)	64.96 (60.12 to 69.81)	64.95 (58.68 to 71.22)
Shrinkage factor (SD)	0.70 (0.15)	0.66 (0.14)

Abbreviations: C-index, concordance index; CRT-AC, chemoradiotherapy followed by adjuvant chemotherapy; IC-CRT, induction chemotherapy followed by concurrent chemoradiotherapy; OS, overall survival; SD, standard deviation.

**TABLE A5:** Characteristic of Nine Example Cases, Including Age, Sex, TNM Staging, Histology, NLR, and BMI, With Predicted 5-Year OS

Case No.	Sex-Age	TNM Stage	Histology	NLR	BMI	5-Year OS (95% CI)	
						CRT-AC	IC-CRT
1	A 54-year-old male	T3N3M0	Undiff	Low	Normal	57.2 (51.2 to 63.9)	39.7 (32.0 to 52.0)
2	A 54-year-old male	T3N3M0	Undiff	High	Overweight	55.8 (49.7 to 62.6)	8.4 (4.3 to 16.3)
3	A 54-year-old male	T3N3M0	Undiff	High	Underweight	49.4 (43.0 to 56.8)	48.7 (40.2 to 59.1)
4	A 56-year-old male	T3N1M0	Undiff	Low	Normal	78.6 (74.9 to 82.4)	67.8 (61.1 to 5.2)
5	A 56-year-old male	T3N1M0	Undiff	High	Overweight	77.8 (73.9 to 81.7)	34.2 (25.6 to 45.6)
6	A 56-year-old male	T3N1M0	Undiff	High	Underweight	73.7 (69.4 to 78.3)	52.6 (44.3 to 62.5)
7	A 58-year-old male	T4N0M0	Undiff	Low	Normal	73.4 (69.1 to 78.0)	64.2 (57.1 to 72.3)
8	A 58-year-old male	T4N0M0	Undiff	High	Overweight	72.4 (67.9 to 77.2)	29.5 (21.2 to 40.9)
9	A 58-year-old male	T4N0M0	Undiff	High	Underweight	67.7 (62.7 to 73.1)	48.1 (39.6 to 58.6)

Abbreviations: CRT-AC, chemoradiotherapy followed by adjuvant chemotherapy; IC-CRT, induction chemotherapy followed by concurrent chemoradiotherapy; N, node; NLR, neutrophil-lymphocyte ratio; M, metastasis; OS, overall survival; T, tumor; Undiff, undifferentiated.
